# Implementation of Virtual Sensors for Monitoring Temperature in Greenhouses Using CFD and Control

**DOI:** 10.3390/s19010060

**Published:** 2018-12-24

**Authors:** Cesar H. Guzmán, José L. Carrera, Héctor A. Durán, Javier Berumen, Arturo A. Ortiz, Omar A. Guirette, Angélica Arroyo, Jorge A. Brizuela, Fabio Gómez, Andrés Blanco, Héctor R. Azcaray, Marlen Hernández

**Affiliations:** 1 Departamento de Ingeniería Mecatrónica, Universidad Politécnica de Zacatecas, Fresnillo 99080, Mexico; jlcarrera20@hotmail.com (J.L.C.); hduran@upz.edu.mx (H.A.D.); jberumen@upz.edu.mx (J.B.); aaortizhernandez@upz.edu.mx (A.A.O); omarguirette@hotmail.com (O.A.G.); angara5@hotmail.com (A.A.); 2 Centro Universitario de los Valles, Universidad de Guadalajara, Ameca 46600, Mexico; jorge.brizuela@valles.udg.mx; 3 Unidad Académica Puerto Vallarta, Instituto Tecnológico José Mario Molina Pasquel y Henríquez, Tecnológico Nacional de México, Puerto Vallarta 48338, Mexico; fabioabelgo@hotmail.com; 4 Centro Nacional de Investigación y Desarrollo Tecnológico (Cenidet), Tecnológico Nacional de México, Cuernavaca 62490, Mexico; andres.blanco@cenidet.edu.mx (A.B.); hector.azcaray.rivera@gmail.com (H.R.A.); 5 Unidad Académica de Matemáticas, Universidad Autónoma de Zacatecas, Zacatecas 98060, Mexico; mar_h2o@hotmail.com

**Keywords:** CFD, greenhouse, monitoring, virtual sensor, temperature control

## Abstract

Virtual sensing is crucial in order to provide feasible and economical alternatives when physical measuring instruments are not available. Developing model-based virtual sensors to calculate real-time information at each targeted location is a complex endeavor in terms of sensing technology. This paper proposes a new approach for model-based virtual sensor development using computational fluid dynamics (CFD) and control. Its main objective is to develop a three-dimensional (3D) real-time simulator using virtual sensors to monitor the temperature in a greenhouse. To conduct this study, a small-scale greenhouse was designed, modeled, and fabricated. The controller was based on the convection heat transfer equation under specific assumptions and conditions. To determine the temperature distribution in the greenhouse, a CFD analysis was conducted. Only one well-calibrated and controlled physical sensor (temperature reference) was enough for the CFD analysis. After processing the result that was obtained from the real sensor output, each virtual sensor had learned the associative transfer function that estimated the output from given input data, resulting in a 3D real-time simulator. This study has demonstrated, for the first time, that CFD analysis and a control strategy can be combined to obtain system models for monitoring the temperature in greenhouses. These findings suggest that, generally, virtual sensing can be applied in large greenhouses for monitoring the temperature using a 3D real-time simulator.

## 1. Introduction

Food production is increasingly being recognized as a serious worldwide concern [1,2]. One solution is the use of greenhouses to protect plants against disease and unexpected climatic events [3,4]. One of the main advantages of greenhouses is the ability to control the optimal temperature for crop cultivation [5,6]. Recently, researchers have shown an increased interest in monitoring greenhouse temperature using different sensor configurations [7], hardware architectures [8,9], and control techniques [10,11]. Currently, monitoring greenhouse temperature is complex in terms of sensing technology [12,13]. Questions have been raised regarding physical sensors’ temperature monitoring efficiency located inside greenhouses. For instance, the implementation of wireless sensor networks (WSN) has demonstrated benefits for crop monitoring [14,15,16,17]. However, the installation of temperature sensors inside the greenhouse increases the cost of engineering and management. There are four important issues to take into consideration before installing a WSN: (a) time-consuming maintenance, (b) need for calibration, (c) natural deterioration, and (d) unexpected failure. Additionally, the direct exposure of constant sunlight and humidity to the sensors may lead to malfunction or damage, which may result in significant economic losses to farmers.

Virtual sensing, also known as soft sensing, has been successfully applied to various processes to provide feasible and economical alternatives when physical measuring instruments are not available [18]. Virtual sensors are software algorithms that use hardware sensors to calculate real-time information at each location of interest [19]. Virtual sensors can be classified into two categories: model-based and data-driven [20]. Model-based virtual sensors use the available measurements and parameters for calculations and they are often implemented through data validation and system models [21]. On the other hand, data-driven virtual sensors base calculations on available historical measurement data obtained either from sensor systems installed temporarily or from detailed estimations. The estimations are typically based on function approximation and regression techniques, such as least squares [22] or neural networks [23], which can be implemented using statistical or machine learning modeling methods.

In many fields of engineering, virtual sensors have been used for various purposes, including noise control [24], distillation columns [25], automotive applications [26], vehicle control [27], gas sensing systems [28], aircraft engines [29], fault detection of nonlinear systems [30], and mobile robots [31]. However, the number of studies that investigate the use of virtual sensors in greenhouses is limited. Davis proposed a technique of adaptive temperature control in a greenhouse using ventilator adjustments [32]. A sensor with variable parameters was fitted to successive samples of aperture and internal temperature data to predict the ventilator adjustments that maintained the required temperature. Piñón et al. presented a scheme for the temperature control of a greenhouse based on data-driven virtual sensors [33]. This study combined feedback linearization and standard linear model predictive control. Speetjens et al. proposed an extended Kalman filter for automatic online estimation and the adaptation of parameters in a physics-based greenhouse model, describing the air temperature and moisture content [34]. Hammed explored the ability of the extended Kalman filter to improve the efficiency of the greenhouse climate controller using the online estimated air temperature and humidity ratio as the controlled variables, instead of the observed noisy states [35].

Recently, computational fluid dynamics (CFD) has been used as an alternative to monitor temperature in greenhouses [36,37,38]. Kittas et al. developed a simple model to estimate the air temperature in a fan-ventilated greenhouse [39]. To create the model, measurements of the ventilation rate and climate variables inside and outside the greenhouse (air temperature and solar radiation) were taken in a multi-span fan-ventilated greenhouse. Molina-Aiz et al. investigated the efficiency of both, finite-element and finite-volume, discretization methods for the simulation of two-dimensional incompressible turbulent flow in naturally ventilated greenhouses [40]. The simulation was optimized using different parameters to analyze the effectiveness of the experimental data in three different types of greenhouses. Tong et al. numerically predicted the time-dependent temperature distribution inside a greenhouse while using CFD [41]. Deltour proposed the Gembloux greenhouse dynamic model of heat and mass transfer to describe its inner climatic variations [42]. This classical one-dimensional thermodynamic model calculated the balance of dynamic energy of air, vegetation, and soil. Trigui et al. introduced an algorithm that is based on heat balances to predict the CO_2_ level, temperature, and relative humidity for maintaining the ideal climate conditions in greenhouses [43]. Longo and Gasparella studied thermal balance, focusing on the energy performance of the greenhouse air conditioning system [44].

To date, research has focused on physical sensors and CFD for monitoring the temperature in greenhouses, but virtual sensing has not been investigated in detail. Sánchez-Molina et al. proposed virtual sensors for designing irrigation controllers in greenhouses [45]. The authors used estimators to provide continuous transpiration measurements. For tomato crops, the availability of estimators allowed for the design of automatic irrigation and fertilization schemes in greenhouses, which minimize the dispensed water while fulfilling crop needs. Roldán et al. described the design, construction, and validation of a mobile sensory platform for greenhouse monitoring [46]. This system consisted of a virtual sensor system inside a quadrotor, which measured the temperature, humidity, luminosity, and CO_2_ concentration while plotting maps of these variables. Pawlowski et al. presented the monitoring and controlling of the greenhouse environment [47]. They described how the greenhouse climate control was represented as an event-based system combined with WSN and virtual sensors, where low-frequency dynamics variables were controlled, and the control actions were mainly calculated with reference to events produced by external disturbances.

This paper proposes a new approach for model-based virtual sensor development based on CFD and control. The main objective of this research is to develop a three-dimensional (3D) real-time simulator based on virtual sensors for monitoring temperature in a greenhouse. The purpose of this study is to provide a starting point for further investigations, using a small-scale greenhouse, featuring practical development in large-scale greenhouses. Thus, the main contribution of this study is to present a new alternative for monitoring temperature in greenhouses by combining CFD and control. It will contribute to the field of virtual sensing by exploring new ways to calculate measurements and parameters using CFD-based system models. To the best of our knowledge, this has not been done before in this field. This approach represents an innovative, feasible, and economical alternative for monitoring the temperature in greenhouses. Due to practical constraints, this paper will not provide experimental results from a real greenhouse. Nevertheless, the methodology that is described here can be extrapolated effectively. The presentation of this paper is structured, as follows. Section 2 contains a description of the materials and methods used in this study, and Section 3 discusses its results. Finally, Section 4 concludes the paper and suggests the scope for future works.

## 2. Materials and Methods

**Figure 1:**
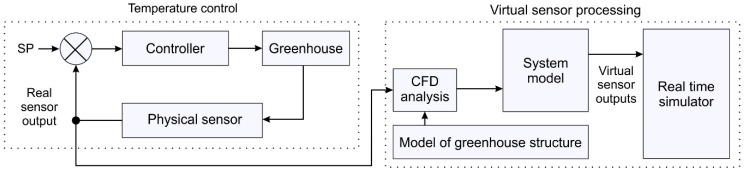
System concept.

A model-based virtual sensor needs a real sensor output and a system model—or transfer function—to create a new sensor; see Figure 1. The aim of this study was divided into two objectives: temperature control and virtual sensor processing. To achieve the first objective, a greenhouse was designed, modeled, and fabricated; the control strategy was proposed to establish real sensor output. To achieve the second objective, the real sensor output was used as an input signal in the virtual sensor processing stage, resulting in a 3D real-time simulator that was based on virtual sensors for temperature monitoring. To establish the greenhouse system model, both the real sensor output and the CFD analysis were used.

### 2.1. Greenhouse Design

**Figure 2:**
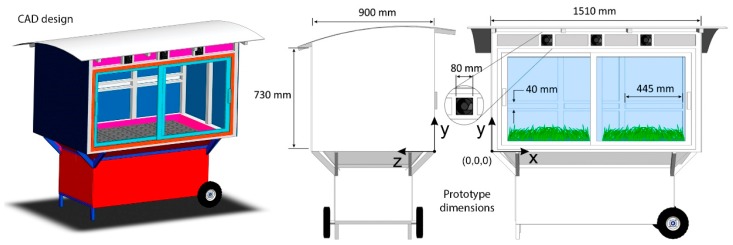
Greenhouse design.

To conduct this study, a greenhouse was designed. For experimental purposes, the greenhouse´s overall dimensions were selected as 1510 mm (length) × 900 mm (width) × 730 mm (height). Figure 2 shows the greenhouse design that included all necessary elements—inlets, outlets, heaters, and fans—for supporting temperature control. The electric resistance heaters were located behind the fans. These fans introduced air into the greenhouse.

### 2.2. Modeling

**Figure 3:**
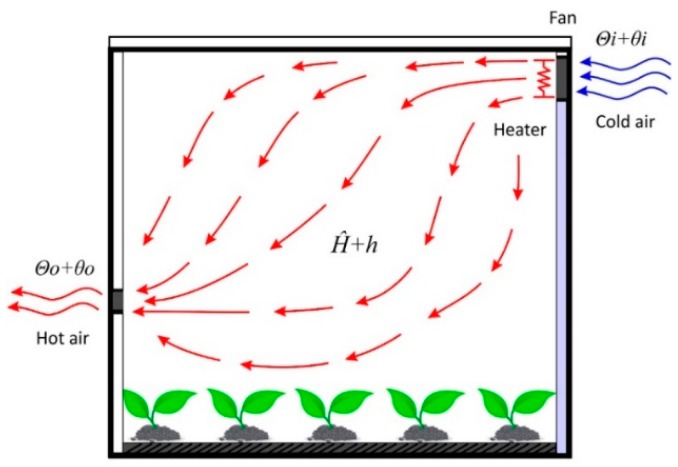
Schematic model of the greenhouse air heating system.

Controllers are always related to mathematical models [48,49]. For this study, the controller was proposed based on the convection heat transfer equation. Certain assumptions and conditions were assumed, such as heat loss being considered as negligible, there was no heat storage in the insulation, and the air flow in the greenhouse was evenly distributed. Figure 3 shows the schematic model of the greenhouse air heating system, which is explained below.

The convection heat transfer equation is given by

(1) q=HAΔθ

where q is the heat flow rate, H is the convection coefficient, A is the normal area of the heat flow, and Δθ is the temperature difference. For this equation, the air flow temperature is kept constant and the heat input can suddenly change from $\hat{H}$ to $\hat{H}+h$. The inlet and outlet air temperature, then, changes from $\Theta_{i}$ to $\Theta_{i}+\theta_{i}$ and from $\Theta_{o}$ to $\Theta_{o}+\theta_{o}$, respectively. For this case, h, C, and R are defined, as follows:

(2) $$h=Gc\theta_{o}$$

(3) C=Mc

(4) $$R=\frac{1}{Gc}$$

The equation that describes the thermodynamic behavior of the greenhouse is given by the following equation:

(5) $$C\frac{d\theta_{o}}{dt}=h+Gc\left(\theta_{i}-\theta_{o}\right)$$

which may be rewritten, as follows:

(6) $$RC\frac{d\theta_{o}}{dt}+\theta_{o}=\theta_{i}+Rh$$

where c is the specific heat of air, R is the thermal resistance, $\hat{H}$ is the steady-state heat input, h is the heat input change, $\Theta_{i}$ is the steady-state inlet air temperature, $\theta_{i}$ is the inlet air temperature change, $\Theta_{o}$ is the steady-state outlet air temperature, $\theta_{o}$ is the outlet air temperature change, M is the mass of air, G is the mass flow rate of air, and C is the thermal capacitance of air. The controller design has been explained below.

If the temperature of the inlet air fluctuates and acts as a disturbance, to achieve temperature control, a controller is necessary to adjust the output of the heaters to compensate for these fluctuations and disturbances. The thermodynamic equation can be rewritten as state variables to determine the greenhouse behavior. This state-space description may be rewritten, as follows:

(7) $$x_{1}=\theta_{o},\;x_{2}={\dot{\theta }}_{o}$$

(8) $${\dot{x}}_{1}=x_{2}$$

(9) $$x_{2}=\frac{h}{C}+\frac{1}{RC}\left(\theta_{i}-x_{1}\right)$$

It is important to mention that the greenhouse’s behavior is completely controllable and it involves two control variables: h and $\theta_{i}$. To control the temperature in the greenhouse, a proportional controller was used due to its simplicity. Introducing the controller as a new control input, the thermodynamic equation can be rewritten, as follows:

(10) $${\dot{\theta }}_{o}=\frac{h}{C}+\frac{1}{RC}\left(\theta_{i}-\theta_{o}\right)=v$$

(11) $$v=-k_{p}\left(\theta_{o}-\theta_{o}^{*}\right)$$

(12) $${\dot{\theta }}_{o}+k_{p}\left(\theta_{o}-\theta_{o}^{*}\right)=0$$

where v is the proportional controller and $k_{p}$ is the controller gain.

Defining the tracking error as *e*, the controller gain can be obtained using the Laplace transformation, as follows:

(13) $$e=\theta_{o}-\theta_{o}^{*}$$

(14) $$\dot{e}+k_{p}e=0$$

(15) $$s+k_{p}=0,\;k_{p}>0$$

### 2.3. Governing Equations

To find the temperature distribution in the greenhouse, a CFD analysis must be conducted. Heat and mass transfer are accomplished by solving the governing equations of fluid dynamics—continuity, momentum, and energy. In this study, the fluid was assumed to be incompressible. The governing equations are expressed below:

Continuity

(16) $$\nabla \cdot \left(\rho \vec{u}\right)=0$$

Momentum

(17) $$\nabla \cdot \left(\rho \vec{u}\vec{u}\right)=-\nabla p+\nabla \cdot \left(\overset{=}{\tau }\right)+\rho \vec{g}$$

(18) $$\overset{=}{\tau }=\mu \left[\left(\left(\nabla \vec{u}\right)\;+\;{\left(\nabla \vec{u}\right)}^{T}\right)-\frac{2}{3}\nabla \cdot \vec{u}\overset{=}{I}\right]$$

Energy

(19) $$\nabla \cdot \left(\rho \vec{u}H\right)=\nabla \cdot \left(\frac{k_{t}}{C_{p}}\nabla H\right)+S_{h}$$

(20) $$H=\overset{T}{\underset{T_{0}}{\int }}C_{p}dT$$

where $\vec{u}$ is the velocity vector, ρ is the density, p is the pressure, $\vec{g}$ is the gravitational acceleration, μ is the viscosity, H is the enthalpy, $k_{t}$ is the thermal conductivity, $C_{p}$ is the specific heat, $S_{h}$ is a source term, T is the temperature, $\overset{=}{I}$ is the identity matrix, and $\overset{=}{\tau }$ is the stress tensor.

### 2.4. Greenhouse Fabrication

There were 27 sensors distributed in the greenhouse—one real and 26 virtual. The distribution of these sensors is shown in Table 1. The purpose of developing a small-scale greenhouse was to conduct controlled experiments without unexpected disturbances. Since several physical sensors over the greenhouse do not represent a substantial contribution to the state of the art, the experimental design included only one physical sensor and 26 virtual sensors. This study is practical, because only one physical sensor has been used to reduce the time-consuming maintenance to farmers.

**Figure 4:**
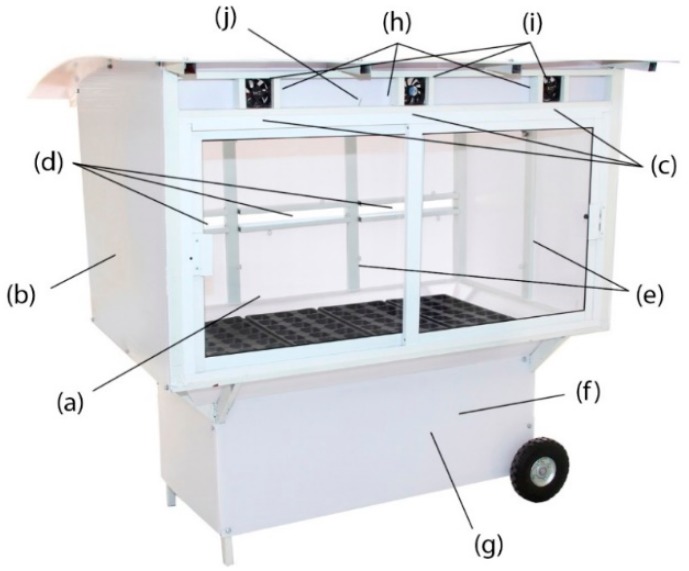
Greenhouse for experimental tests.

The proposed design was then fabricated for experimental tests, as shown in Figure 4. The main structure of the greenhouse was made of steel and protected with a thin layer of white paint (a). The greenhouse walls were made of 3 mm thick polycarbonate (b). Three air inlets were installed in the front of the greenhouse (c). Three air outlets were installed in the back of the greenhouse (d). Two glass sliding doors were installed in the front of the greenhouse (e). One acquisition card (NI USB 6009) was selected to support the analog and digital data conversion (f). The sampling time of this card was set to 100 ms. Two drivers were manufactured to control the current to the fans and the electric resistance heaters (g). Three electric resistance heaters were installed to heat the inlet air (h). Three fans were installed to bring air into the greenhouse (i). One real temperature sensor was installed (j) and the resolution was 14-bit with an accuracy of ±0.3 °C.

## 3. Results

### 3.1. Temperature Control

**Figure 5:**
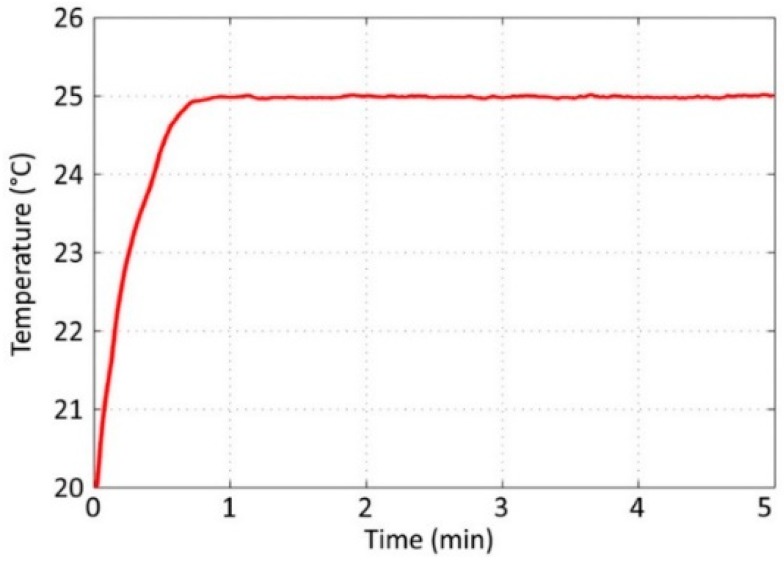
Result obtained from the real sensor output.

**Figure 6:**
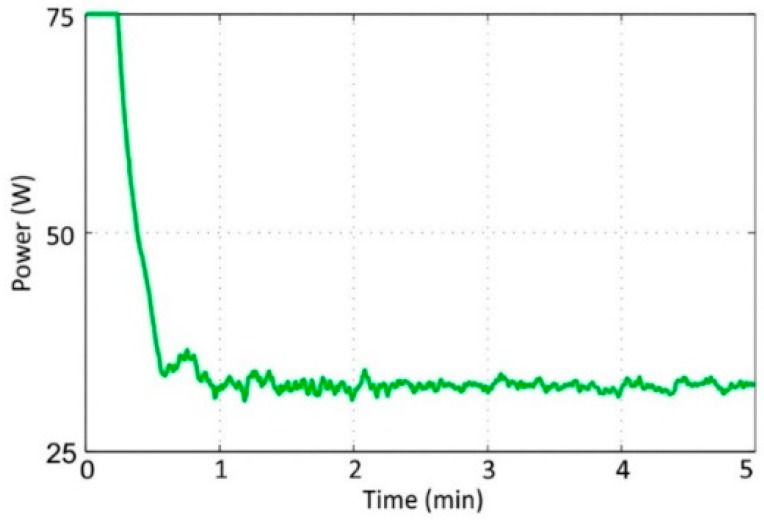
Controller output.

To assess the performance of the controller, the set point was established at 25 °C and the experiment time was set as five minutes. The temperature was monitored and recorded with a sampling time of one second. Figure 5 shows the result that was obtained from the real sensor output. The implemented controller reached the set point in less than 45 seconds and maintained the desired temperature. It demonstrated an accuracy of ±0.3 °C. The controller output was limited to 75 W to avoid overheating the system’s hardware; see Figure 6. The controller output used 75 W to reach the set point and 30 W to maintain steady state. These experimental results indicated that the proposed controller was adequate for the next step—virtual sensor processing.

### 3.2. CFD Simulation

To predict the temperature distribution based on the greenhouse structure, the governing equations were solved numerically using a CFD software package and a desktop PC (Intel® Core ™ i7 CPU at 2.67 GHz with 8 GB of RAM).

#### 3.2.1. Boundary Conditions

For this CFD analysis, the boundary conditions were defined as follows:

- Walls: A no-slip shear condition was selected; the wall temperature was set to 10.00 °C
- Inlet: The inlet air velocity was set to 0.5 m/s
- Outlet: The atmospheric pressure was set to 101 kPa and the outlet temperature was set to 10.00 °C
- Chamber: The dimensions were 1510 mm × 900 mm × 730 mm
- Inlets: The dimensions were 80 mm × 80 mm
- Outlets: The dimensions were 445 mm × 40 mm

#### 3.2.2. Grid Convergence Study

**Figure 7:**
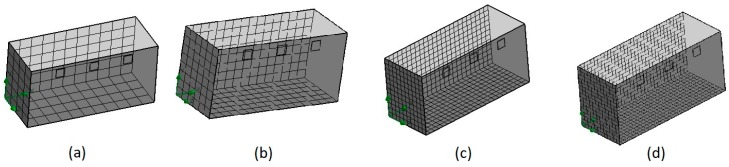
Mesh sensitivity analysis. (**a**) 5070, (**b**) 7760, (**c**) 17,242, (**d**) 19,258.

**Figure 8:**
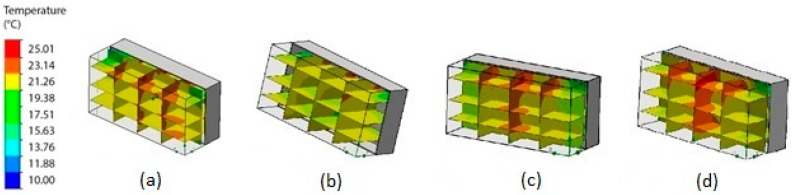
Temperature distribution of the greenhouse using different mesh sizes. (**a**) 5070, (**b**) 7760, (**c**) 17,242, (**d**) 19,258.

Establishing grid convergence is crucial in any numerical study [50]. The results must be guaranteed using a grid convergence study, which is essential to verify that the governing equations are being solved correctly and that the solution is not sensitive to the grid resolution. Thus, a mesh sensitivity analysis was conducted to find the size and number of cells that were suitable for this study. Four simulations using different mesh sizes were conducted. Figure 7 demonstrates the results of the mesh sensitivity analysis. The relationship between the size and number of cells is shown in Table 2. Figure 8 shows the temperature distribution of the greenhouse using different mesh sizes. It is observed that the temperature distribution was more uniform using 17,242 and 19,258 cells, and the highest temperature zone was located at the center of the greenhouse. Using more than 17,242 cells did not exhibit a significant difference. Thus, this mesh was used for virtual sensor processing.

#### 3.2.3. Inlet Air Velocity

**Figure 9:**
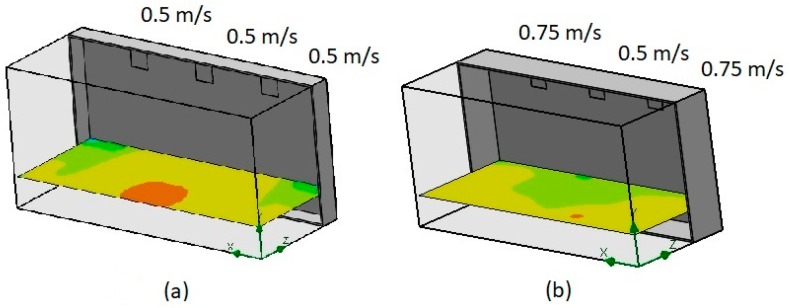
Temperature analysis considering different inlet air velocities. (**a**) using the same inlet air speeds, (**b**) using different inlet air speeds.

Three different inlet air speeds were proposed. Figure 9a shows the temperature contours using the same inlet air speeds (0.5 m/s). Figure 9b shows the temperature contours using different inlet air speeds (0.75, 0.5, and 0.75 m/s). A comparison of both results indicated a difference in the temperature distribution. In this study, the inlet air speeds were considered to be constant.

#### 3.2.4. Temperature Distribution

**Figure 10:**
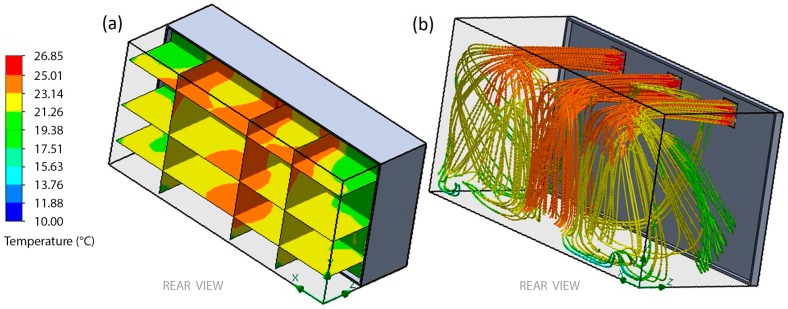
Temperature distribution in the greenhouse. (**a**) section views, (**b**) flow trajectories.

According to the result obtained from the mesh analysis, the CFD analysis was conducted with reference to the temperature control. The inlet temperature was set to 25.01 °C. Three vertical and horizontal section views were the output from the CFD simulation. Figure 10a shows the temperature distribution in the greenhouse. Figure 10b shows the flow trajectories of temperature. These results revealed a lot of new information. First, the temperatures vary from 25.01 °C to 19.38 °C. Second, the majority of the temperatures in the greenhouse were concentrated in the yellow zones. Third, the green zones show heat loss due to convective heat transfer to the lateral walls.

### 3.3. Temperature Monitoring

Previous studies have conducted temperature analysis of greenhouses using CFD in detail. The validation of CFD models has been done before in the state of the art using physical sensors as reference points inside a greenhouse. The physical sensors were located at different positions in order to compare the experimental and simulation results. These studies demonstrated significant similarities. Therefore, this study combined CFD and control theory. Thus, the contribution of this study is not its experimental validation, but it is its new approach of using virtual sensors. The 3D temperature distribution of the greenhouse was calculated in detail without having to install new indoor sensors; see Table 3.

**Figure 11:**
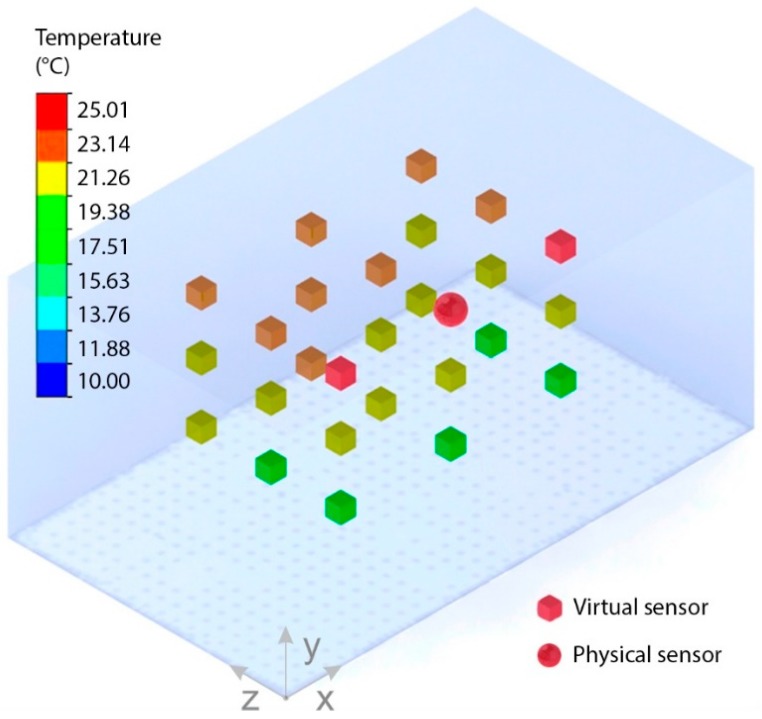
Three-dimensional (3D) real-time simulator.

The task at this stage was to find a relationship model between the real sensor output and the CFD analysis. At the end of the virtual sensor processing, each virtual sensor had learned the associative transfer function that links its input readings to estimate an output. Following this, all sensors—real and virtual—were included in a 3D real-time simulator, as shown in Figure 11. As demonstrated, only one well-calibrated and controlled physical sensor (temperature reference) was enough for the CFD analysis and the real-time 3D simulator. In fact, the size of the greenhouse was not a limitation in demonstrating the implementation of the virtual sensors.

## 4. Conclusions and Future Work

This paper presented a new approach for model-based virtual sensor development based on CFD and control. The present study was designed to create virtual sensors though temperature control and virtual sensor processing. A synergistic combination of sensors, mechanics, electronics, modelling, control, thermal science, systems, instrumentation, CFD, and mechatronics was conducted to provide an alternative in the field of greenhouse design and management. Simulation results were presented to show the temperature distribution of the greenhouse. These findings suggest that, generally, virtual sensing can be applied in large greenhouses for monitoring temperature using a 3D real-time simulator. Possible applications of virtual sensors in commercial greenhouses will be noticed and real-time CFD will appear to develop a promising approach for the very effective and efficient control of the indoor environment.

On the other hand, this study found that virtual sensing enabled monitoring temperatures in the greenhouse while using only one sensor. However, there was no uniform distribution of temperature in the greenhouse. This finding was unexpected and it suggests that the temperature distribution must be improved using different greenhouse architectures—this is an important issue for future research. Therefore, the results greatly depend on where the real sensor is located. Nevertheless, these results were very promising. Virtual sensors may represent a promising solution for temperature monitoring in large-scale greenhouses. In real-world applications, CFD analysis can include more parameters. For the estimation of the temperature in a specific area of an industrial greenhouse, the CFD analysis may combine solar radiation, external temperature, wind speed, and other factors. Further studies will need to take these variables into account. We believe that virtual sensors will be the next step in instrumentation and monitoring, not only in greenhouses but also in aquaponics. The next study will analyze temperature control using virtual sensors instead of real sensors. Furthermore, it will be very interesting to include humidity virtual sensors as well as a virtual hygrometer.

## Figures and Tables

**Table 1:** Greenhouse sensor distribution.

No.	Type of Sensor	Coordinates X, Y, Z (mm)	No.	Type of Sensor	Coordinates X, Y, Z (mm)	No.	Type of Sensor	Coordinates X, Y, Z (mm)
1	virtual	400, 172, 225	10	virtual	755, 172, 225	19	virtual	1110, 172, 225
2	virtual	400, 172, 450	11	virtual	755, 172, 450	20	virtual	1110, 172, 450
3	virtual	400, 172, 675	12	virtual	755, 172, 675	21	virtual	1110, 172, 675
4	virtual	400, 365, 225	13	virtual	755, 365, 225	22	virtual	1110, 365, 225
5	virtual	400, 365, 450	14	virtual	755, 365, 450	23	virtual	1110, 365, 450
6	virtual	400, 365, 675	15	virtual	755, 365, 675	24	virtual	1110, 365, 675
7	virtual	400, 547, 225	16	real	755, 547, 225	25	virtual	1110, 547, 225
8	virtual	400, 547, 450	17	virtual	755, 547, 450	26	virtual	1110, 547, 450
9	virtual	400, 547, 675	18	virtual	755, 547, 675	27	virtual	1110, 547, 675

**Table 2:** Relation of size and number of cells.

	(a)	(b)	(c)	(d)
Number of cells in X	11	16	24	32
Number of cells in Y	6	8	12	16
Number of cells in Z	4	6	10	12
Total Cells	5070	7760	17,242	19,258
Solid Cells	2154	3344	8270	10,582
Fluid Cells	2916	4416	8972	8676

**Table 3:** Virtual sensor outputs.

No.	Temperature (°C)	No.	Temperature (°C)	No.	Temperature (°C)
1	19.38	10	19.38	19	19.38
2	19.38	11	21.26	20	19.38
3	21.26	12	23.14	21	21.26
4	21.26	13	21.26	22	21.26
5	21.26	14	21.26	23	21.26
6	21.26	15	23.14	24	21.26
7	25.01	16	25.01	25	25.01
8	23.14	17	23.14	26	23.14
9	23.14	18	23.14	27	23.14

## References

[1] Parry M.L., Rosenzweig C., Iglesias A., Livermore M., Fischer G. (2004). Effects of climate change on global food production under SRES emissions and socio-economic scenarios. Glob. Environ. Chang..

[2] Crist E., Mora C., Engelman R. (2017). The interaction of human population, food production, and biodiversity protection. Science.

[3] Hanan J.J. (2017). Greenhouses: Advanced Technology for Protected Horticulture.

[4] Oliveira J., Boaventura-Cunha J., Oliveira P.M. (2017). Automation and control in greenhouses: State-of-the-art and future trends. CONTROLO 2016, Lecture Notes in Electrical Engineering.

[5] Hassanien R.H.E., Li M., Lin W.D. (2016). Advanced applications of solar energy in agricultural greenhouses. Renew. Sustain. Energy Rev..

[6] Kumar K., Tiwari K., Jha M.K. (2009). Design and technology for greenhouse cooling in tropical and subtropical regions: A review. Energy Build..

[7] Shamshiri R., Kalantari F., Ting K., Thorp K.R., Hameed I.A., Weltzien C., Ahmad D., Shad Z.M. (2018). Advances in greenhouse automation and controlled environment agriculture: A transition to plant factories and urban agriculture. Int. J. Agric. Biol. Eng..

[8] Van Beveren P., Bontsema J., Van Straten G., Van Henten E. (2015). Minimal heating and cooling in a modern rose greenhouse. Appl. Energy.

[9] Carvajal-Arango R., Zuluaga-Holguín D., Mejía-Gutiérrez R. (2016). A systems-engineering approach for virtual/real analysis and validation of an automated greenhouse irrigation system. Int. J. Interact. Des. Manuf..

[10] Márquez-Vera M.A., Ramos-Fernández J.C., Cerecero-Natale L.F., Lafont F., Balmat J.-F., Esparza-Villanueva J.I. (2016). Temperature control in a MISO greenhouse by inverting its fuzzy model. Comput. Electron. Agric..

[11] Bennis N., Duplaix J., Enéa G., Haloua M., Youlal H. (2008). Greenhouse climate modelling and robust control. Comput. Electron. Agric..

[12] Mukhopadhyay S.C. (2012). Smart Sensing Technology for Agriculture and Environmental Monitoring.

[13] Ruiz-Garcia L., Lunadei L., Barreiro P., Robla I. (2009). A review of wireless sensor technologies and applications in agriculture and food industry: State of the art and current trends. Sensors.

[14] Akshay C., Karnwal N., Abhfeeth K., Khandelwal R., Govindraju T., Ezhilarasi D., Sujan Y. Wireless sensing and control for precision Greenhouse management. Proceedings of the 2012 Sixth International Conference on Sensing Technology.

[15] Rodriguez S., Gualotuna T., Grilo C. (2017). A System for the monitoring and predicting of data in precision agriculture in a rose greenhouse based on wireless sensor networks. Procedia Comput. Sci..

[16] Ferentinos K.P., Katsoulas N., Tzounis A., Bartzanas T., Kittas C. (2017). Wireless sensor networks for greenhouse climate and plant condition assessment. Biosyst. Eng..

[17] Calderon-Cordova C., Bustamante B., Delgado J., Febres C., Montano V., Saritama C., Ramirez C. Wireless sensor network for real-time monitoring of temperature, humidity and illuminance in an orchid greenhouse. Proceedings of the 13th Iberian Conference on Information Systems and Technologies.

[18] Wilson E. Virtual sensor technology for process optimization. Proceedings of the Symposium on Computers and Controls in the Metals Industry in Iron and Steel Society.

[19] Liu L., Kuo S.M., Zhou M. Virtual sensing techniques and their applications. Proceedings of the International Conference on Networking, Sensing and Control.

[20] Lin B., Recke B., Knudsen J.K., Jorgensen S.B. (2007). A systematic approach for soft sensor development. Comput. Chem. Eng..

[21] Prasad V., Schley M., Russo L.P., Bequette B.W. (2002). Product property and production rate control of styrene polymerization. J. Process Control.

[22] Park S., Han C. (2000). A nonlinear soft sensor based on multivariate smoothing procedure for quality estimation in distillation columns. Comput. Chem. Eng..

[23] Radhakrishnan V., Mohamed A. (2000). Neural networks for the identification and control of blast furnace hot metal quality. J. Process Control.

[24] Costantini R., Susstrunk S. (2004). Virtual sensor design. Sensors and Camera Systems for Scientific, Industrial, and Digital Photography Applications.

[25] Kano M., Ogawa M. (2010). The state of the art in chemical process control in Japan: Good practice and questionnaire survey. J. Process Control.

[26] Wenzel T., Burnham K., Blundell M., Williams R. (2007). Kalman filter as a virtual sensor: Applied to automotive stability systems. Trans. Inst. Meas. Control.

[27] Canale M., Fagiano L., Ruiz F., Signorile M.C. A study on the use of virtual sensors in vehicle control. Proceedings of the 47th IEEE Conference on Decision and Control.

[28] Ankara Z., Kammerer T., Gramm A., Schütze A. (2004). Low power virtual sensor array based on a micromachined gas sensor for fast discrimination between H_2_, CO and relative humidity. Sens. Actuators B Chem..

[29] Xue W., Guo Y.-Q., Zhang X.-D. (2008). Application of a bank of Kalman filters and a robust Kalman filter for aircraft engine sensor/actuator fault diagnosis. Int. J. Innov. Comput. Inf. Control.

[30] Fekih A., Xu H., Chowdhury F.N. (2007). Neural networks based system identification techniques for model based fault detection of nonlinear systems. Int. J. Innovat. Comput. Inf. Control.

[31] Bellas F., Becerra J., Santos J., Duro R.J. Applying synaptic delays for virtual sensing and actuation in mobile robots. Proceedings of the International Joint Conference on Neural Networks.

[32] Davis P. (1984). A technique of adaptive control of the temperature in a greenhouse using ventilator adjustments. J. Agric. Eng. Res..

[33] Piñón S., Camacho E., Kuchen B., Peña M. (2005). Constrained predictive control of a greenhouse. Comput. Electron. Agric..

[34] Speetjens S., Stigter J., Van Straten G. (2009). Towards an adaptive model for greenhouse control. Comput. Electron. Agric..

[35] Hameed I.A. (2010). Using the extended Kalman filter to improve the efficiency of greenhouse climate control. Int. J. Innovat. Comput. Inf. Control.

[36] De la Torre-Gea G., Soto-Zarazúa G.M., López-Crúz I., Torres-Pacheco I., Rico-García E. (2011). Computational fluid dynamics in greenhouses: A review. Afr. J. Biotechnol..

[37] Alvarez-Sánchez E., Leyva-Retureta G., Portilla-Flores E., López-Velázquez A. (2014). Evaluation of thermal behavior for an asymmetric greenhouse by means of dynamic simulations. Dyna.

[38] Reichrath S., Davies T.W. (2002). Using CFD to model the internal climate of greenhouses: Past, present and future. Agronomie.

[39] Kittas C., Karamanis M., Katsoulas N. (2005). Air temperature regime in a forced ventilated greenhouse with rose crop. Energy Build..

[40] Molina-Aiz F., Fatnassi H., Boulard T., Roy J., Valera D. (2010). Comparison of finite element and finite volume methods for simulation of natural ventilation in greenhouses. Comput. Electron. Agric..

[41] Tong G., Christopher D., Li B. (2009). Numerical modelling of temperature variations in a Chinese solar greenhouse. Comput. Electron. Agric..

[42] Deltour J., De Halleux D., Nijskens J., Coutisse S., Nisen A. Dynamic modelling of heat and mass transfer in greenhouses. Proceedings of the Symposium Greenhouse Climate and Its Control.

[43] Trigui M., Barrington S., Gauthier L. (2001). A strategy for greenhouse climate control, Part II: Model validation. J. Agric. Eng. Res..

[44] Longo G.A., Gasparella A. (2012). Comparative experimental analysis and modelling of a flower greenhouse equipped with a desiccant system. Appl. Therm. Eng..

[45] Sánchez-Molina J.A., Rodríguez F., Guzmán J.L., Arahal M.R. (2012). Virtual sensors for designing irrigation controllers in greenhouses. Sensors.

[46] Roldán J.J., Joosen G., Sanz D., del Cerro J., Barrientos A. (2015). Mini-UAV based sensory system for measuring environmental variables in greenhouses. Sensors.

[47] Pawlowski A., Guzman J.L., Rodríguez F., Berenguel M., Sánchez J., Dormido S. (2009). Simulation of greenhouse climate monitoring and control with wireless sensor network and event-based control. Sensors.

[48] Guzmán-Valdivia C.H., Carrera-Escobedo J.L., García-Ruíz M.A., Ortíz-Rivera A., Désiga-Orenday O. (2016). Design, development and control of a portable laboratory for the chili drying process study. Mechatronics.

[49] Guzmán C.H., Blanco A., Brizuela J.A., Gómez F.A. (2017). Robust control of a hip–joint rehabilitation robot. Biomed. Signal Process. Control.

[50] Carrera-Escobedo J.L., Ortíz-Rivera A., Guzmán-Valdivia C.H., García-Ruíz M.A., Désiga-Orenday O. (2018). CFD analysis for improving temperature distribution in a chili dryer. Therm. Sci..

